# Effect of dietary cellulose supplementation on gut barrier function and apoptosis in a murine model of endotoxemia

**DOI:** 10.1371/journal.pone.0224838

**Published:** 2019-12-02

**Authors:** Valentina Di Caro, Alicia M. Alcamo, Jessica L. Cummings, Robert S. B. Clark, Elizabeth A. Novak, Kevin P. Mollen, Michael J. Morowitz, Rajesh K. Aneja

**Affiliations:** 1 Department of Critical Care Medicine, University of Pittsburgh School of Medicine, Pittsburgh, PA, United States of America; 2 Department of Pediatrics, University of Pittsburgh School of Medicine, Pittsburgh, PA, United States of America; 3 Division of Pediatric Critical Care Medicine, UPMC Children’s Hospital of Pittsburgh, Pittsburgh, PA, United States of America; 4 Department of Surgery, University of Pittsburgh School of Medicine, Pittsburgh, PA, United States of America; 5 Division of Pediatric General and Thoracic Surgery, UPMC Children’s Hospital of Pittsburgh, Pittsburgh, PA, United States of America; National Institute for Agronomic Research, FRANCE

## Abstract

The gut plays a vital role in critical illness, and alterations in the gut structure and function have been reported in endotoxemia and sepsis models. Previously, we have demonstrated a novel link between the diet-induced alteration of the gut microbiome with cellulose and improved outcomes in sepsis. As compared to mice receiving basal fiber (BF) diet, mice that were fed a non-fermentable high fiber (HF) diet demonstrated significant improvement in survival and decreased organ injury in both cecal-ligation and puncture (CLP) and endotoxin sepsis models. To understand if the benefit conferred by HF diet extends to the gut structure and function, we hypothesized that HF diet would be associated with a reduction in sepsis-induced gut epithelial loss and permeability in mice. We demonstrate that the use of dietary cellulose decreased LPS-mediated intestinal hyperpermeability and protected the gut from apoptosis. Furthermore, we noted a significant increase in epithelial cell proliferation, as evidenced by an increase in the percentage of bromodeoxyuridine-positive cells in HF fed mice as compared to BF fed mice. Thus, the use of HF diet is a simple and effective tool that confers benefit in a murine model of sepsis, and understanding the intricate relationship between the epithelial barrier, gut microbiota, and diet will open-up additional therapeutic avenues for the treatment of gut dysfunction in critical illness.

## Introduction

Under basal conditions, the components of the intestinal microenvironment (intestinal epithelium, microbiome, and host immune system) act in concert to maintain a symbiotic, mutually beneficial relationship [1]. Critical illness impairs intestinal integrity by a) alteration of protein junction complexes that govern the paracellular barrier, and b) direct cell damage due to an increase in epithelial apoptosis and a decrease in epithelial proliferation [2]. In addition, diseases like sepsis result in a breakdown of the alliance mentioned above and transform the microbiome, resulting in a loss of microbial diversity, diminution of pro-health commensal microbes, and an increase in the abundance of pathogenic bacteria [3, 4]. The presence of these dominant harmful pathogens within fecal samples has been identified as a risk factor for subsequent infections caused by that same organism [5, 6] and can contribute to metabolic, immune, and neurocognitive disturbances in the critically-ill host [7]. After the onset of sepsis, these disturbed microbial communities can lead to immune exhaustion and a loss of T-helper cells, thereby setting the stage for infections by the dominant pathogens [8].

Previously, we have demonstrated a novel link between the diet-induced alteration of the gut microbiome with cellulose and improved outcomes in murine models of sepsis [9]. In general, fiber represents a group of carbohydrates or carbohydrate-containing compounds that are neither digested nor absorbed in the small intestine [10, 11]. During chronic or intermittent dietary fiber deficiency, the gut microbiota resorts to host-secreted mucus glycoproteins as a nutrient source, leading to erosion of the colonic mucus barrier [12]. Based on the fermentation potential, the quantity and type of fibers will differentially modulate the evolution of the intestinal microbiome. Specifically, we noted that as compared to the basal fiber (BF) diet, mice that were fed a non-fermentable high-fiber (HF) diet demonstrated significant improvement in survival and decreased organ injury in both cecal-ligation and puncture (CLP) and endotoxin sepsis models [9]. Notably, we found that murine fecal samples collected after 2 weeks of an HF diet were highly enriched with *Akkermansia*, a genus associated with metabolic health, and taxa from the family *Lachnospiraceae*, which includes beneficial anaerobes commonly found in the healthy colon [9].

The gut has been characterized as the “motor” of multiple organ dysfunction syndrome (MODS) in critical-illness, a testament to the vital role it plays in pathogenesis [13]. It has been postulated that dysregulated crosstalk between the gut epithelium, immune system, and endogenous microflora [14] leads to the development of MODS [2, 15]. Based on our previous findings that the use of HF diet improved survival in murine sepsis that was associated with alteration of the gut microbiome, a logical extension of our study was to investigate if the HF diet-mediated benefits extend to the gut structure and function. In this study, we hypothesize that the HF diet is associated with a reduction in gut epithelial loss and amelioration of the LPS-induced intestinal permeability.

## Material and methods

### Animal care and animal use

C57BL/6 male mice were purchased from The Jackson Laboratory (Bar Harbor, ME, USA). A total of 220 mice were used in this study. All animal experiments were performed with approval from the University of Pittsburgh IACUC, and care of the animals was in accordance with the National Institutes of Health guidelines for animal treatment. Twenty-four hours after arrival, mice (4–6 weeks old) were randomized to receive chemically defined BF or HF diets (Dyets, Bethlehem, PA) with fiber concentration of 5% and 30%, respectively. As per the manufacturer, the source of fiber in the mouse chow is alpha-cellulose derived from natural vegetable fibers. Apart from the difference in the fiber concentration, the diets were otherwise similar (supplementary table) [9]. The number of mice housed was 4 per cage. Mouse weights as well as the amount of food eaten per cage was documented daily. After 2 weeks on the assigned diet, sepsis was induced in BF and HF diet mice by intraperitoneal (IP) injection of endotoxin (LPS serotype 055B5, 30 mg/kg) (Sigma-Aldrich, St. Louis, MO, USA). Additional controls included mice on BF and HF diets that were not administered LPS and will henceforth be referred to as BC and HC, respectively. Animals that showed signs of distress, e.g., unable or unwilling to walk around their cage, hunched posture, loss of body weight, or piloerection, were removed from the study and euthanized. Mice were anesthetized with isoflurane to collect blood via terminal cardiac puncture, and cecal tissues at 6, 24, or 72 h after LPS administration.

**Fig 1:**
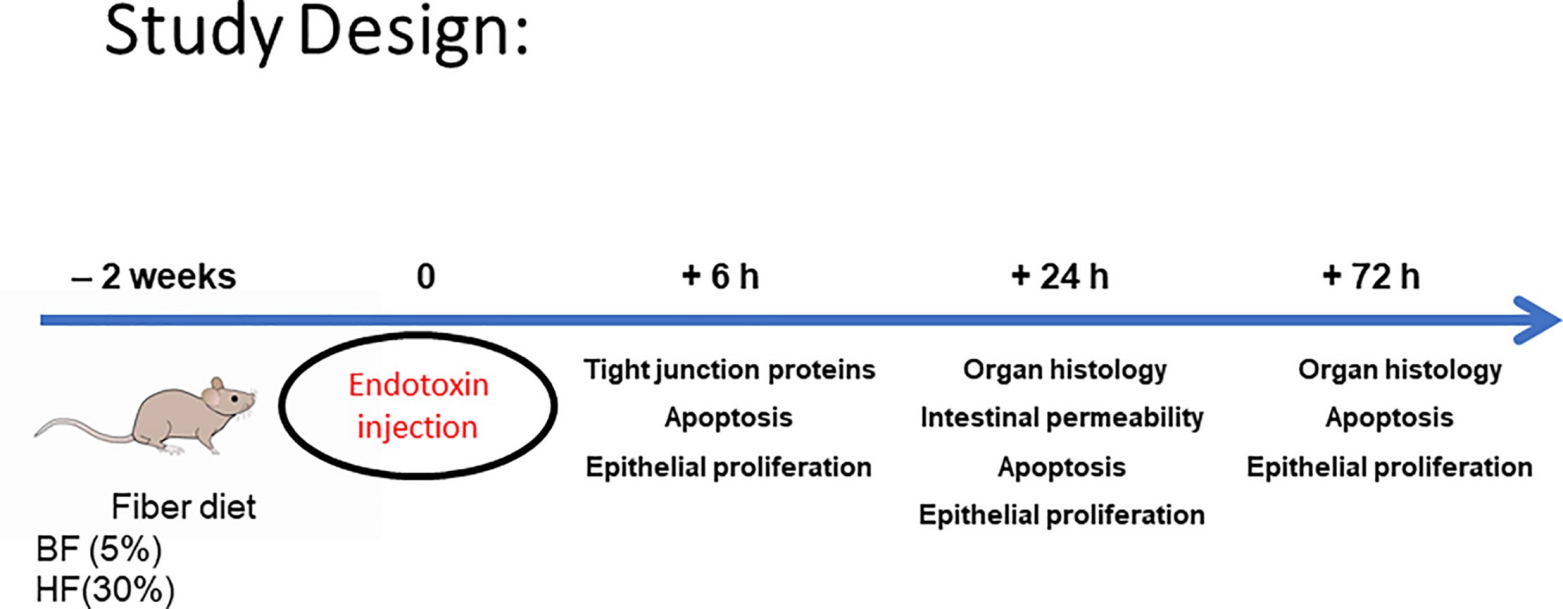
Illustration of the study timeline.

Plasma and tissue samples were stored frozen at −80°C until further analysis (Fig 1). The number of mice used per experiment is specified in the figure legends.

### Histology

Cecum sections at 24 or 72 h post endotoxin injection were fixed in water-free Methanol-Carnoy’s fixative (Thermo Fisher Scientific, Waltham, MA, USA) and embedded in paraffin. Tissue sections were cut at 5 μm and stained with haematoxylin and eosin (H&E). Slices were analyzed under light microscope.

### Tunnel staining

Cecum sections at 6, 24 or 72 h post endotoxin injection were deparaffinized, rehydrated and washed with PBS. Sections were then permeabilized using 20 μg/ml ProteinaseK for 30 min at 37°C (Sigma-Aldrich, St Louis MO, USA) and incubated with the Tunel reaction mixture from the “*in situ* detection kit” from Roche (Basel, Switzerland) following the manufacture instructions. Cell death was assessed by quantifying Tunel positive cells in 5 to 10 mice counting 3 slides per mouse under confocal microscope.

### Protein preparation and western blot

Total protein extracts from cecal tissues at 6h were obtained using M–PER protein extraction reagents as appropriate (Thermo Fisher Scientific, Waltham, MA, USA). Protein concentration was determined by the BCA Protein Assay Kit (Thermo Fisher Scientific, Waltham, MA, USA) and standardized to bovine serum albumin. Protein lysates were separated in a 10% SDS-PAGE gel, electro-transferred onto a PVDF membrane (BioRad Biotechnology, Hercules, CA, USA) and incubated with specific primary antibodies (Caspase3, Bax and Bcl-xl, Santa Cruz Biotechnology, Dallas TX, USA; Occludin, Claudin-1, Invitrogen Carlsbad, CA, USA, β-actin, Sigma-Aldrich, St. Louis, MO, USA) followed by incubation with a horseradish peroxidase (HRP)-conjugated secondary antibody (HRP-conjugated Goat anti Rabbit IgG, HRP-conjugated Rabbit anti Mouse IgG, HRP-conjugated Rabbit anti Goat IgG Jackson ImmunoResearch Labs, West Grove, PA, USA). Protein–antibody complexes were visualized by enhanced chemiluminescence with an ECL system (GE Healthcare, Little Chalfont, UK). For densitometry analysis, fold induction was calculated by dividing the normalized density from each sample by the density from the control sample.

### Intestinal permeability

After 2 weeks of the dietary intervention, mice were injected with endotoxin. Mice were then gavaged with 0.5 ml of fluorescein isothiocyanate-conjugated (FITC)-dextran (FD-4) (440 mg/kg) (Sigma-Aldrich St. Louis, MO, USA) 4 hours prior to the 24 h sacrifice. At the time of sacrifice, blood was collected and centrifuged at 13000 rpm at 4°C for 5 minutes. Fifty μl of plasma was then diluted with an equivalent volume of PBS (pH 7.4), and the concentration of FD-4 was determined using fluorospectrometry at an excitation wavelength of 470 nm and an emission wavelength of 515 nm (Spectra MAX M2, Molecular Devices, San Jose, CA, USA). Serially diluted samples were used as standards, and all samples were run in triplicate.

### Bromodeoxyuridine staining for cell proliferation

Mice received an IP injection of Bromodeoxyuridine (BrdU) 4 h prior to sacrifice. Cecal tissues, at 6, 24 and 72 h post endotoxin injection, were collected and fixed in paraformaldehyde (PFA). At the time of the staining, sections were deparaffinized, rehydrated and washed with water. The antigens were retrieved by microwave heating the sections in 0.01M citric buffer pH 6. Sections were gently rinse in water and incubated 1h at R.T. in 2M HCL, washed in PBS and blocked with 1% BSA donkey serum solution for 1h at R.T. Samples were then incubated with anti-BrdU Ab (Thermo Fisher Scientific, Hampton, NH, USA) overnight at 4°C.

The following day, sections were incubated 1h at R.T. with a fluorescent secondary antibody (Life technologies, Carlsbad, CA, USA) and counterstained with DAPI (Thermo Fisher Scientific, Hampton, NH, USA). Proliferation was assessed by quantifying BrdU positive cells in 5–6 animals counting 3 slides per mouse under confocal microscope.

### Statistical analyses

Statistical analyses were performed using GraphPad Prism version 7·0 software (San Diego, CA). Data are presented as mean ± standard error of the mean (SEM) derived from at least an n = 6 independent mice; where * indicates the presence of a significant difference when *P* ≤ 0.05 between HF and BF diet mice as determined by ANOVA or Student’s *t*-test.

## Results

### Cellulose rich diet does not alter cecal histomorphology

**Fig 2:**
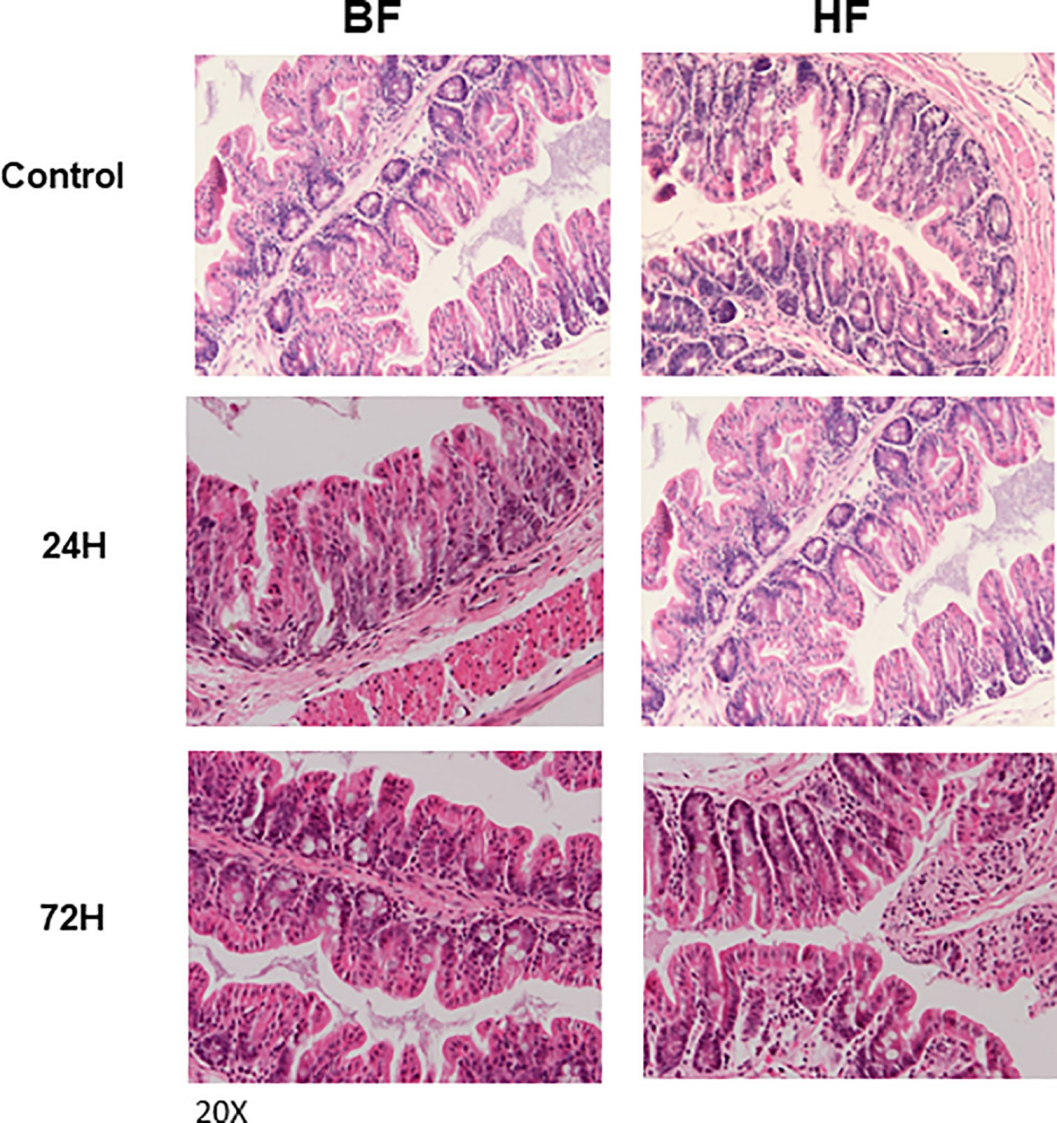
Relative preservation of gut epithelium in BF and HF diet mice that were administered LPS. H&E staining of intestinal tissues from controls, BF and HF diet mice 24 and 72 h post LPS injection. Images are representative of at least 8 different mice. Magnification 20X.

Mice were fed HF or BF diet for two weeks and were sacrificed at 24 or 72 h after LPS administration. Histologic analysis of the cecum demonstrated a normal gross structure of the crypts with minimal submucosal inflammatory cell infiltration in both BF and HF diet mice (Fig 2).

### HF diet ameliorates LPS induced intestinal permeability in mice

**Fig 3:**
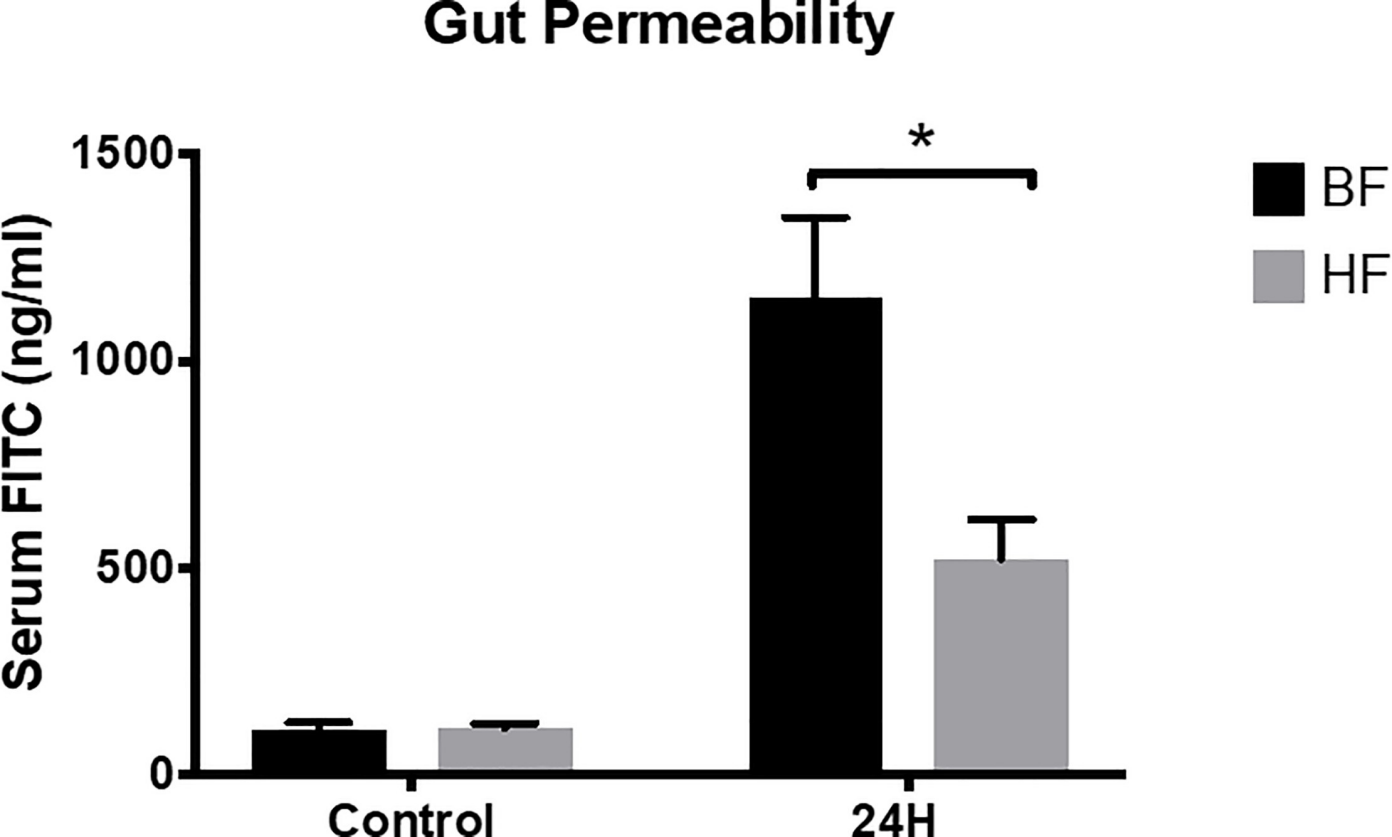
Decreased gut permeability in HF diet mice subjected to endotoxemia. Intestinal permeability at 24 h post-LPS injection was assessed by measuring levels of (FITC)-dextran in blood samples of BF, HF as well as control mice. Each data point represents the mean ± S.E.M. of 10 mice for each group (* indicates the presence of a significant difference when *P* ≤ 0.05 between BF and HF diet mice as determined by ANOVA, Tukey’s multiple comparisons).

We wanted to test if alterations in the HF diet conferred protection on the intestinal barrier. Intestinal permeability, a measurable function of the intestinal barrier, was assessed by administering FITC-dextran. LPS injection induced an approximately 10-fold increase in the intestinal permeability of BF diet mice as compared to control at 24h (1151 ± 196 *vs*. 111 ± 15 ng/ml; *P* = 0.005). In contrast, mice that were fed HF diet demonstrated a significant reduction in LPS-induced intestinal permeability as compared to BF fed mice (523 ± 94 *vs*. 1151 ± 196 ng/ml; *P* = 0.02) (Fig 3). Taken together, we have shown that mice on the HF diet subjected to endotoxin demonstrated a decrease in cecal permeability as compared to mice on BF diet with relative preservation of the intestinal architecture in both groups.

### Effect of HF diet on the expression of intestinal tight junction proteins

**Fig 4:**
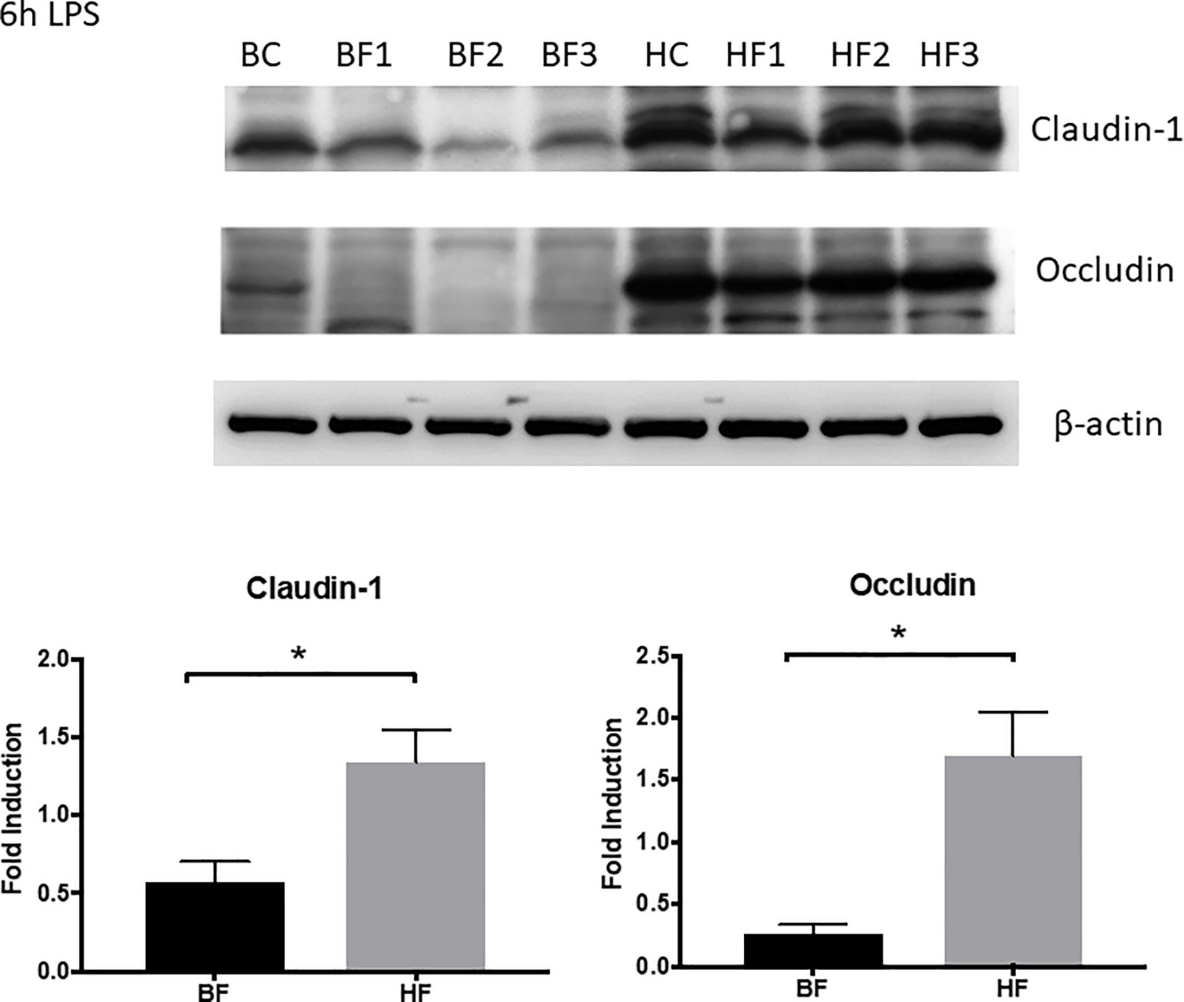
LPS administration demonstrated a significant decrease in the protein expression of both claudin-1 and occludin in BF fed mice as compared to control mice. Representative autoradiograph of Western blot analysis for claudin-1 and occludin in intestinal protein extracts. BC and HC are representative bands from control mice who received the respective diet but did not receive LPS injection. Lanes labeled BF and HF are representative of diet mice 6 h post LPS injection. The radiographs shown are representative of 3 independent experiments. β-actin is shown as a control for equal protein loading in each lane. The graph represents the relative band densities normalized to the basal expression for BC, HC mice (* indicates the presence of a significant difference when *P* ≤ 0.05 between BF and HF diet mice exposed to LPS as determined by t-test).

To understand the contribution of tight junction proteins in the amelioration of the intestinal barrier dysfunction, we next measured the protein expression of claudins and occludin by immunoblotting. As shown in Fig 4, LPS administration demonstrated a significant decrease in the protein expression of both claudin-1 and occludin in BF fed mice after LPS exposure as compared to BC mice (*P* ≤ 0.05). Similarly, the protein expression of claudin 1 and occludin was higher in HF diet mice when compared to BF diet mice after LPS administration (*P* ≤ 0.05).

### High cellulose intake protects the gut from apoptosis during endotoxemia

**Fig 5:**
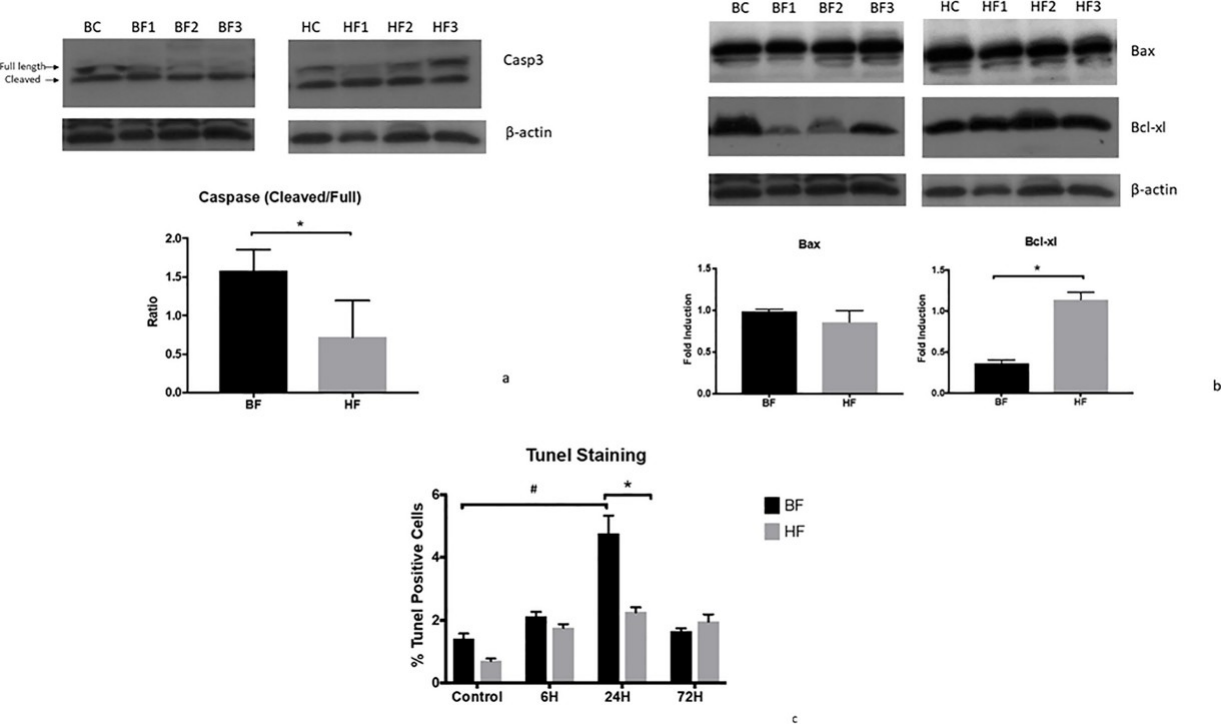
HF diet in mice is associated with a reduction in LPS-mediated intestinal apoptosis. Representative autoradiograph of Western blot analysis of A) Casp-3, B) Bax and Bcl-xl in intestinal protein extracts from BF and HF diet mice 6 h post LPS injection. The radiographs shown are representative of 3 independent experiments. β-actin is shown as a control for equal protein loading in each lane. All three proteins have been stained in the same western and shown here with the same β-actin controls. The graph represents the relative band densities normalized to the basal expression for BC, HC mice. (* indicates the presence of a significant difference when *P* ≤ 0.05 between BF and HF diet mice exposed to LPS as determined by t-test). C) TUNEL assay was used to determine apoptosis in the intestinal epithelium. The graph illustrates the percentage of cell death in BF and HF diet mice 6, 24 and 72 h after LPS injection. Each data point represents the mean ± S.E.M. of 10 mice for each group (*indicates the presence of a significant difference when *P* ≤ 0.05 between BF and HF diet mice as determined by ANOVA, Tukey’s multiple comparisons).

To further elucidate the possible mechanisms of intestinal barrier function preservation in HF diet mice and the central role of gut apoptosis in pathophysiology of multiple diseases [16, 17], we evaluated a number of molecular events that play crucial roles in apoptotic signaling pathways, such as cleavage of caspase 3, expression of Bax and Bcl-xl. LPS cleaves caspase-3 from its inactive form, pro-caspase 3 (32 kDa) to its 17-kDa active form, thereby activating the caspase-mediated death signaling pathways in intestinal tissue. To confirm the enhanced enzymatic activity of caspase-3, cecal lysates were immunoblotted with the anti-caspase-3 antibody. As evident in Fig 5A, 6h after LPS treatment, we noted the cleavage of caspase-3 in BF diet mice as compared to control and HF mice (Fig 5A).

Decreasing intestinal epithelial cell death is associated with improved survival in murine sepsis; hence, our next logical step was to determine if the use of HF diet led to a reduction in intestinal epithelial cell apoptosis. Cecal tissue proteins from each group were extracted 6 h after LPS injection. Western blot was used to determine the levels of apoptotic markers, Bax, and Bcl-xl. Analysis of the anti-apoptotic marker Bcl-xl also showed a significant decrease in BF diet mice after just 6 h of endotoxemia induction (*P* ≤ 0.05). Surprisingly, we did not observe any changes in the expression level of the pro-apoptotic markers Bax in HF diet mice as compared to BF diet mice (Fig 5B).

We then confirmed the anti-apoptotic action of HF diet by evaluating its effect on another marker of apoptosis DNA fragmentation. Using the TUNEL assay, we noted that in comparison to BC mice, BF diet mice demonstrated a significant increase in the apoptotic cell population at 24 h after LPS administration (4.7 ± 0.56 *vs*. 1.4 ± 0.14%; *P* = 0.0033). In contrast, there was a minimal increase in the intestinal apoptotic rate in HF diet-fed mice (2.2 ± 0.15 vs. 0.7± 0.07%; *P* = 0.4). The number of apoptotic cells is comparable in BF and HF diet mice at 72 h post-LPS administration (Fig 5C). Taken together, these findings suggest that the use of HF diet in mice is associated with a reduction in LPS-mediated intestinal apoptosis and associated intestinal permeability as compared to mice on BF diet.

### HF diet increase epithelial cell proliferation in the gut

**Fig 6:**
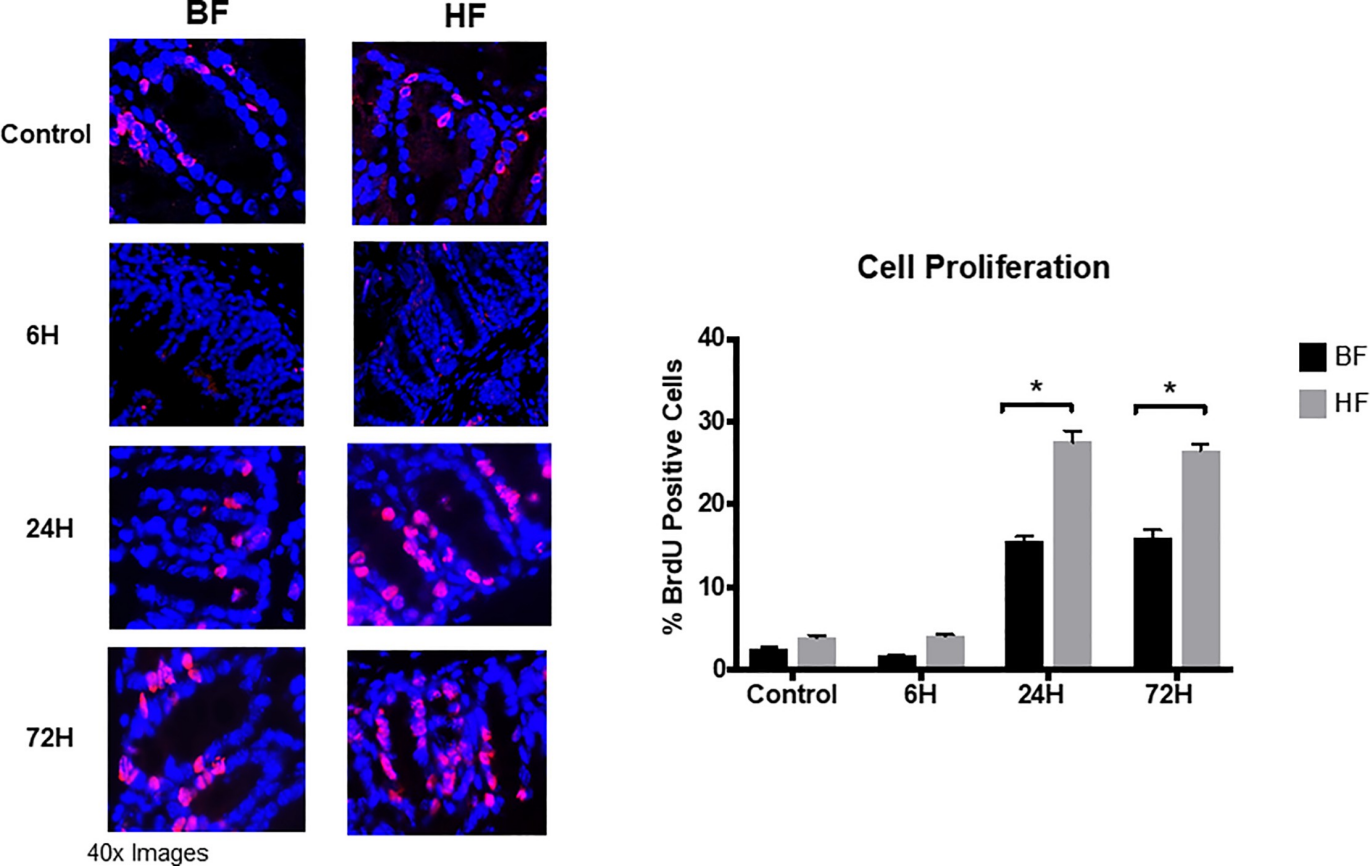
HF diet supplementation is associated with an early increase in intestinal epithelial cell proliferation. Representative images of BrdU stained cecal sections are shown for time points 6, 24 and 72 h after LPS injection. Each data point represents the mean ± S.E.M. of 6 mice for each group (* indicates the presence of a significant difference when P ≤ 0.05 between BF and HF diet mice as determined by ANOVA, Tukey’s multiple comparisons).

To determine whether the use of HF diet modulates the sepsis-induced crypt proliferation, mice fed HF, or BF diet were subjected to endotoxin administration. At 6h after LPS injection, we noted a similar percentage of BrdU-positive cells in both BF and HF diet mice compared to respective control mice. However, 24h post sepsis induction, we observed a significant increase in intestinal proliferation evidenced by an increase in the percentage of BrdU positive cells in HF diet mice compared to BF diet mice (27.5 ± 1.3 *vs*. 15.6 ± 0.5%, *P* < 005). Furthermore, similar rates of proliferation were noted in HF and BF diet mice at 72 h post LPS injection (26.5 ± 0.7 *vs*. 16 ± 0.8%; *P* < 0.05) (Fig 6). Taken together, these data suggest that the use of HF diet decreases gut epithelial apoptosis and is accompanied by an early increase in intestinal epithelial cell proliferation in response to LPS injection within 24 h.

## Discussion

We have previously demonstrated in two different murine models of sepsis, cecal ligation and puncture (CLP) and endotoxemia, that alterations in the microbiome following high cellulose diet supplementation confers survival protection and reduces the systemic pro-inflammatory response. This reduction in pro-inflammatory response can be ascribed to the decrease in cytokines as well as the number and activation of immune cells as splenic macrophages, dendritic cells, and T cells [18]. Mice on the HF diet demonstrated sustained changes in the gut microbiota, notably an enrichment for *Akkermansia*. *Akkermansia*, an anaerobic organism, is a commonly observed member of the human and rodent gut microbiome, and its abundance in humans has been inversely correlated with body weight (32) and inflammatory activity in inflammatory bowel disease. There were three significant findings in our study. First, the use of dietary cellulose decreases LPS-mediated cecal hyperpermeability. Second, remarkably, the use of the HF diet protects the cecum from apoptosis during endotoxemia. Third, we noticed a significant increase in epithelial cell proliferation, as evidenced by an increase in the percentage of BrdU-positive cells in HF fed mice as compared to BF fed mice. Each of these observations merits further discussion.

The initial theories about the relationship between the gut and critical illness suggested that hyperpermeability resulted in bacterial translocation into the systemic circulation; however, the reality is significantly more complex than was hypothesized [13] [19, 20]. Changes in intestinal permeability have been demonstrated in both sepsis and noninfectious models of critical illness [21]. Similar to other studies, we noted increased intestinal permeability at 24 h after endotoxin injection. Altered intestinal permeability is associated with dysbiosis, mucus layer alterations, and epithelial damage, and result in the translocation of luminal contents to the inner layers of the intestinal wall [22]. We have previously demonstrated that the protective effects of cellulose supplementation are microbiota-dependent, such that animals receiving cellulose supplementation were partially protected against systemic inflammation and death in two murine models of sepsis—an effect that is abrogated in the presence of antibiotics [9]. Our data suggest that high fiber diets, in addition to their effect on the host-microbiome, also strengthen the epithelial barrier by modulating the tight junction proteins, thereby decreasing permeability and ameliorating the inflammatory state.

The regulation of normal intestinal mucosal barrier function depends on the proper assembly of tight junctions between adjacent epithelial cells, and previously it has been demonstrated that intestinal hyperpermeability is associated with alteration in numerous claudin isoforms, occludin and ZO-1 [23–25]. Another study noted alteration in claudin-1, -2, -5, JAM-A, and occludin in septic mice while all other tight junction proteins were unchanged [15]. We focused on two tight junctions that have been previously demonstrated to be modulated in two different models of sepsis, cecal ligation and puncture (CLP) and *Pseudomonas aeruginosa* pneumonia. [15, 25]. Although our model for sepsis was different as we used IP injections of LPS to induce sepsis, our results are similar to other studies cited above as there was a notable decrease in claudin-1 and occludin expression in BF mice. However, the noteworthy feature is the significant increase in the cecal expression of “sealing proteins,” claudin-1 and occludin, in HF diet mice as compared to BF diet mice, as this suggests that both proteins may potentially play a role in improving barrier function in sepsis.

All elements of the gut, such as the epithelium, the immune system, and the microbiome, are impacted by critical illness and can, in turn, propagate a pathologic host response [2, 14, 26] leading to the development of MODS [2, 15]. The high constitutive rate of proliferation in the intestinal epithelium and its delicate balance with cell senescence and loss of terminally differentiated cells is well appreciated [27]- however, this relationship is significantly altered in animal models of critical illness and sepsis [16, 28]. For example, in a murine *Pseudomonas aeruginosa* pneumonia model, a time-dependent decrease in gut epithelial proliferation that was coupled with a simultaneous increase in crypt apoptosis has been previously demonstrated [16]. The time course of apoptosis, as characterized by caspase 3 cleavage, is similar to previous studies, where active caspase 3 was noted to be elevated 4 h after bacterial injection and peaked at 12 h [17]. Previously, it has been shown that transgenic mice that overexpress Bcl-2, member of the same family of BCL-xl, had a partial restoration in their proliferative capacity compared with septic wild-type littermates, with a decrease in the number of M-phase cells to basal levels [16]. Another striking finding in this study is that in addition to a decrease in the gut epithelial apoptosis noted in HF diet mice, we observed a significant increase in intestinal proliferation that was demonstratable as early as 24 h after LPS, and persisted at 72 h in HF diet mice as compared to BF diet mice. Thus, while we could anticipate some decrease in gut epithelial apoptosis due to the increased expression of the anti-apoptotic protein Bcl-xl, we did not expect an early increase in epithelial proliferation mediated by the use of HF diet.

The concept of using diet to modulate the gut microbiome to achieve a favorable outcome is not new. However, it is only within the last decade that alterations in the gut microbiome were thought to be associated with immunopathology in several autoimmune and inflammatory diseases, as well as altered susceptibility to infectious diseases [29–31]. Thus, there has been a paradigm shift in medicine from “the germ theory of disease” to the current understanding that the host and its prokaryotic residents exist in a carefully negotiated truce, with compromises and benefits that go both ways [32–36]. A current approach to manipulation of the microbiome is to foster the growth of “beneficial” bacteria by using probiotics. Probiotic trials in some disease states hold promise, although the optimal probiotic prescription is unknown and may vary by disease state. We now provide evidence that dietary supplementation of cellulose improves outcome and is associated with alteration of the gut microbiome in a murine model of sepsis [9]. Fiber has long been recognized for its nutritional value, and as per the last iteration of the Dietary Reference Intakes (DRI), the daily value for fiber is ~14g/1000 Kcal (70–80% insoluble and 20–30% soluble). The novelty of our work was the utilization of cellulose, an insoluble non-fermentable fiber that is a significant component of the vegetarian diet, as it is present in most plant tissues. In contrast to fermentable fiber, where the inflammatory benefits are secondary to the production of short-chain fatty acids, the benefits of cellulose are due to the transformation it brings to the intestinal microbial composition.

In conclusion, our findings suggest that the use of the HF diet can be a simple and effective tool that can decrease gut permeability by its effect on the tight junction proteins and decreasing LPS mediated apoptosis and increasing intestinal epithelial proliferation. Understanding the intricate relationship between the epithelial barrier, gut microbiota, and diet will open up additional therapeutic avenues for the treatment of gut dysfunction in critical illness. Future work involves mechanistic microbiota studies to transplant *Akkermansia* in gnotobiotic mice to quantify the modulatory role of the gut microbiome on the gut barrier function.

## Supporting information

S1 Fig BF and HF diet composition. (PDF)
