# Supplementary material for: Effect of dietary cellulose supplementation on gut barrier function and apoptosis in a murine model of endotoxemia
Source: PLoS One. 2019 Dec 2;14(12):e0224838. doi: 10.1371/journal.pone.0224838 (PMC6886840; doi:10.1371/journal.pone.0224838)
Supplement: S1 Fig — (PDF) [file pone.0224838.s001.pdf]

Appendix A

Diet Composition

Ingredient	HF Diet (g/Kg)	HF Diet (%)	BF Diet (g/Kg)	BF Diet (%)
Casein	200	20	200	20
L-Cystine	3	0.3	3	0.3
Sucrose	100	10	100	10
Cornstarch	147	14.7	397.4	39.74
Dyetrose	132	13.2	132	13.2
Soybean Oil	70	7	70	7
t-Butylhydroquinone	0.014	0.014	0.014	0.014
Cellulose	300	30	50	5
Mineral Mix #210025	35	3.5	35	3.5
Vitamin Mix # 310025	10	1	10	1
Choline Bitartrate	2.5	0.25	2.5	0.25
Calories (kcal/kg)	2860		3760	

HF- High Fiber (30%)

BF-Basic Fiber (5%)
